# Cryoablation and radiofrequency ablation during mitral valve surgery for rheumatic mitral valve disease: a retrospective cohort study

**DOI:** 10.3389/fcvm.2025.1659310

**Published:** 2026-01-22

**Authors:** Zhanar Nurbay, Auyeskhan Dzhumabekov, Roza Kuanishbekova, Rustem Tuleutayev, Nurzhan Musrepov

**Affiliations:** 1 SCE City Cardiology Center, Almaty, Kazakhstan; 2 Kazakhstan Medical University KSPH, Almaty, Kazakhstan; 3 JSC Research Institute of Cardiology and Internal Diseases, Almaty, Kazakhstan

**Keywords:** atrial fibrillation, cardiac surgery, cryoablation, radiofrequency ablation, rheumatic mitral valve disease

## Abstract

**Background and aims:**

Atrial fibrillation (AF) frequently accompanies rheumatic mitral valve disease (MVD) and adversely affects postoperative outcomes. Radiofrequency ablation (RFA) and cryoablation are commonly used during mitral valve surgery, but their comparative impact on atrial remodeling in this population remains uncertain.

**Methods:**

This retrospective cohort included 100 patients with rheumatic MVD and persistent AF who underwent mitral valve surgery with concomitant cryoablation (*n* = 50) or RFA (*n* = 50) between June 2020 and June 2024 at centers in the Almaty region, Kazakhstan. Clinical and echocardiographic parameters were assessed preoperatively, within 48 h postoperatively, and at 6 ± 2 months.

**Results:**

Cryoablation was associated with greater left atrial (LA) volume reduction immediately and at follow-up (both *p* < 0.001). Multiple linear regression identified ablation modality as the only independent predictor of LA volume reduction (*β* = 27.9 mL, *p* < 0.0001), whereas duration of rheumatic disease, BMI, EuroSCORE II, and AF recurrence were not significant. At follow-up, the reduction in right atrial short-axis diameter was smaller after cryoablation (*p* = 0.049), and stroke volume declined less compared with RFA (–1.2 ± 17.3 mL vs. −7.3 ± 15.8 mL; *p* = 0.006). Cardiopulmonary bypass time, aortic cross-clamp time, and postoperative symptom improvement were comparable between groups. Freedom from AF during follow-up was also similar (log-rank *p* = 0.52).

**Conclusions:**

In patients with persistent AF and rheumatic MVD undergoing mitral valve surgery, cryoablation was associated with more pronounced early atrial reverse remodeling and better preservation of stroke volume compared with RFA, without differences in operative efficiency or short-term safety. These findings should be considered hypothesis-generating, and prospective randomized studies with standardized lesion sets are required to confirm modality-specific effects.

## 1 Introduction

Atrial fibrillation (AF) is the most prevalent clinically relevant cardiac arrhythmia worldwide (1). AF affects over 59 million individuals globally, and prevalence is projected to double or triple by 2050 (2, 3). This rise is significant for countries like Kazakhstan, where demographic shifts are similar to those observed in Europe, with an increasing proportion of elderly individuals (4). This demographic trend is especially crucial as age remains the strongest risk factor for AF (5).

AF is associated with a significantly higher incidence of ischemic stroke and heart failure, contributing to morbidity and mortality. Without timely diagnosis and appropriate intervention, these complications place a strain on health and social care systems (2, 6). Although diagnostic methods such as electrocardiography (ECG), Holter monitoring, and portable wearable devices have improved AF detection globally, access remains limited among underrepresented and resource-constrained populations (7). In Kazakhstan, diagnosis is largely restricted to ECG and Holter methods, which may underestimate the true prevalence (8, 9). Moreover, despite guidelines for early initiation of oral anticoagulants in patients to prevent thrombotic complications, concerns about bleeding risks often lead to underuse or inappropriate substitution with antiplatelet drugs (10).

The situation is further complicated in patients with rheumatic mitral valve disease, as among them AF occurs more frequently. Surgical ablation, performed together with valve procedures, offers an opportunity to restore sinus rhythm and improve long-term outcomes. Two primary approaches that are currently used for this purpose are radiofrequency ablation (RFA) and cryoablation (11, 12). RFA uses high-frequency electrical current to create linear transmural lesions that interrupt abnormal conduction pathways, whereas cryoablation achieves the same effect through localized freezing of atrial tissue (11, 13).

Several studies have demonstrated that both RFA and cryoablation are effective in restoring and maintaining sinus rhythm in patients with persistent atrial fibrillation undergoing surgical ablation (14, 15). In surgical settings, cryoablation provides highly consistent and reproducible lesions with minimal collateral injury, owing to its uniform lesion depth and reduced dependence on operator technique, making it an attractive and safe option for intraoperative ablation (16–18). Some surgical series and reviews indicate comparable long-term safety profiles for cryoablation and radiofrequency energy when AF ablation is performed during cardiac surgery (19–21), though high-quality comparative data remain limited, particularly in rheumatic mitral valve disease.

This retrospective study aims to compare the effectiveness and safety of RFA and cryoablation for AF in patients with rheumatic mitral valve disease undergoing mitral valve surgery. Given the ethical and logistical constraints of randomizing surgical ablation modality, a retrospective cohort design was used to evaluate outcomes in a real-world clinical setting. The study aims to provide evidence to guide surgical decision-making in this high-risk and understudied patient group. Because lesion-set geometry and procedural strategy may differ between energy modalities in routine practice, this study focuses on real-world outcomes rather than isolated biophysical effects of ablation energy.

## 2 Methods

### 2.1 Study population and study design

This retrospective cohort included consecutive patients treated at collaborating centers in the Almaty region, Kazakhstan, between June 2020 and June 2024. Because this was a retrospective analysis of existing medical records, no formal sample-size calculation was performed. Records were reviewed chronologically, and, before outcome analysis, an *a priori* accrual rule prespecified inclusion of two sequential consecutive series of eligible patients: the first 50 who underwent cryoablation and the next 50 who underwent radiofrequency ablation (RFA). The energy source in each case was determined by the operating surgeon, based on institutional device availability; no randomization or study-driven assignment occurred.

All included patients had persistent atrial fibrillation with a documented duration of ≤3 years prior to surgery, as confirmed by electrocardiographic and clinical records. Inclusion criteria were rheumatic mitral valve disease undergoing open-heart mitral valve repair or replacement with concomitant surgical ablation and availability of 6 ± 2-month follow-up. Exclusion criteria were ischemic (non-rheumatic) mitral regurgitation, decompensated heart failure with marked chamber dilatation, incomplete clinical records, long-standing persistent AF (>3 years), or absence of 6 ± 2-month follow-up.

**Figure 1:**
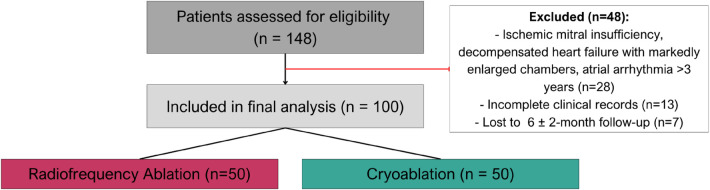
Participant flow and allocation. Patients assessed for eligibility (*n* = 148). Excluded (*n* = 48): ischemic mitral insufficiency or decompensated heart failure with markedly enlarged chambers (*n* = 28), incomplete clinical records (*n* = 13), and no 6 ± 2-month follow-up (*n* = 7). Included in final analysis (*n* = 100). Allocation was chronological and nonrandom: first 50 eligible consecutive cryoablation cases and next 50 eligible consecutive radiofrequency cases, based on surgeon's discretion and device availability.

Patient screening, exclusions, and chronological allocation are shown in Figure 1. The study was approved by the local institutional review board (IRB-19-2023), and trial registration was not applicable.

This sequential accrual strategy reflected changes in institutional equipment availability rather than a study-driven treatment assignment.

### 2.2 Data collection

Patient demographics, comorbidities, and procedural details were extracted from electronic health records using a standardized data collection form. Two trained coordinators independently abstracted all key variables. Any discrepancies were reviewed and resolved by a third, independent reviewer. All available data were complete for the variables analyzed, so no imputation methods were applied.

As a retrospective study, selection bias is possible due to the non-random assignment of ablation modality. To minimize this, data were collected from multiple high-volume centers using standardized surgical and assessment protocols. All analyses were performed blinded to group assignment during data abstraction.

### 2.3 Surgical ablation strategy and lesion set

Cryoablation and radiofrequency ablation (RFA) were performed concomitantly with open-heart valve replacement surgery according to the standard operating protocols of each participating center. The choice of ablation modality was determined by institutional practice and surgeon preference at the time of surgery; patients were not randomized due to the retrospective study design. All included patients had persistent atrial fibrillation confirmed by electrocardiographic and clinical records. Because lesion-set geometry differed between cryoablation and radiofrequency strategies, the present comparison evaluates real-world procedural approaches rather than isolated energy effects.

Surgical ablation was performed using either cryothermal or radiofrequency energy. In the cryoablation group, lesions were created using the AtriCure system, which provides controlled cooling and reproducible formation of transmural lesions. In the RFA group, ablation was performed according to the principles of the Cox–Maze IV procedure using a monopolar Cardioblate™ electrode (Medtronic, USA).

Although both strategies were based on contemporary surgical AF treatment concepts derived from the Cox–Maze procedure, the detailed configuration of lesion lines differed between cryoablation and RFA in accordance with energy-specific surgical protocols.

#### 2.3.1 Left atrial ablation

##### 2.3.1.1 Cryoablation group

Pulmonary vein isolation was achieved by creating continuous circumferential lesions encircling the ostia of the right and left pulmonary veins. Cryolesions were applied on the posterior wall of the left atrium at the sites of venous insertion, maintaining a safe distance from the esophagus.

In addition, a longitudinal posterior left atrial wall line was created along the interatrial groove toward the mitral valve to interrupt potential macro-reentry circuits. A mitral isthmus line was applied from the inferior segment of the right pulmonary vein encirclement toward the lateral mitral annulus to prevent peri-mitral atrial flutter.

##### 2.3.1.2 RFA group

In the RFA group, left atrial ablation was performed as a box-lesion set to achieve complete electrical isolation of the posterior left atrial wall. This configuration included a superior connecting line between the right and left superior pulmonary veins, an inferior connecting line between the right and left inferior pulmonary veins, and right- and left-sided encircling lines around the pulmonary vein ostia, resulting in full posterior wall isolation.

An additional lesion was created from the superior margin of the box-lesion to the base of the left atrial appendage. A mitral isthmus line was applied from the inferior margin of the box-lesion to the posterolateral portion of the mitral annulus (P2–P3 region).

#### 2.3.2 Right atrial ablation

##### 2.3.2.1 Cryoablation group

A cavotricuspid isthmus line was created along the anatomical isthmus between the inferior vena cava and the tricuspid annulus. In selected patients, additional right atrial free-wall lesions were applied based on anatomical features and concomitant pathology.

##### 2.3.2.2 RFA group

Right atrial ablation in the RFA group consisted of the following lesion lines:

1. a cavocaval line from the superior vena cava to the inferior vena cava along the posterior right atrial wall;
2. a cavotricuspid isthmus line from the inferior vena cava to the tricuspid annulus (5–6 o'clock position);
3. a line from the superior vena cava or anterior right atrial wall to the base of the right atrial appendage;
4. a connecting line from the inferoposterior right atrial wall to the ostium of the coronary sinus.

#### 2.3.3 Left atrial appendage management

Surgical management of the left atrial appendage (LAA) was performed using two techniques: epicardial clipping with an AtriClip device and surgical suturing, depending on intraoperative findings and surgeon preference, and was applied in both the cryoablation and RFA groups.

#### 2.3.4 Lesion set completeness and verification

Lesion completeness was assessed intraoperatively using standard criteria, including visual continuity of ablation lines, stable tissue contact, and anchoring to anatomical fibrous structures. Electrophysiological confirmation of conduction block was performed when technically feasible, depending on procedural conditions and rhythm status.

### 2.4 Echocardiographic and ECG assessments

Routine transthoracic echocardiograms and ECGs (with Holter monitoring) were performed according to each center's standard operating protocols. Echo studies were done within one week before surgery, then again within 48 h afterward, and once more at a six month period.

All measurements were conducted according to standardized protocols across all participating centers, and data collection procedures were identical between the cryoablation and RFA groups.

The primary outcome was the change in left atrial (LA) volume from baseline to 6-month follow-up. Secondary outcomes included change in right atrial (RA) diameter, stroke volume, intraoperative parameters (CPB time, ACC time, blood loss), left atrial appendage occlusion technique, and procedural complications.

### 2.5 Six-min walk test

Functional capacity data came from standard 6-min walk tests administered as part of routine follow-up.

### 2.6 Statistical analysis

Analyses were performed in GraphPad Prism v10.4.2. Normality was tested by Shapiro–Wilk (*p* ≥ 0.05 = normal); group comparisons used unpaired *t*-tests or Mann–Whitney *U* as appropriate, and categorical data used *χ*^2^ tests. Freedom from AF recurrence was assessed by Kaplan–Meier with log-rank testing. Multiple linear regression modeled change in LA volume (mL) with prespecified covariates (ablation type, BMI, EuroSCORE II, disease duration, AF recurrence); results are reported as *β*, 95% CI, *p*, *R*^2^, and *F*-statistics. Assumptions were checked via residual diagnostics and variance inflation factors. Two-tailed *p* < 0.05 was considered significant.

### 2.7 Ethical considerations

Ethical approval for the study was obtained from the Ethics Committee of the Kazakhstan Medical University «KSPH» under protocol number № IRB-19-2023. This manuscript follows the STROBE guidelines. A completed checklist is provided in the Supplementary Materials.

## 3 Results

### 3.1 Baseline characteristics

Of 148 patients assessed, 48 were excluded, leaving 100 for analysis: 50 cryoablation and 50 radiofrequency (Figure 1).

The distributions of patients by sex, body mass index (BMI), and disease duration were not significantly different between the cryoablation and RFA groups. The difference in mean disease duration between the two groups was not statistically significant (*p* = 0.691), indicating that baseline duration of rheumatic heart disease was comparable. The preoperative mortality risk, assessed using the EuroSCORE II scale, was similar in the cryoablation and RFA groups (*p* = 0.166), confirming their comparability in terms of surgical risk (Table 1). The complexity category of the operation was assessed on a 10-point scale and was 5 for all patients, corresponding to the average severity of the intervention.

**Table 1:** Baseline characteristics of patients undergoing cryoablation or RFA.

Variable	Total (*n* = 100)	Cryoablation (*n* = 50)	RFA (*n* = 50)
Age (years, mean ± SD)	58.8 ± 12.2	59.5 ± 11.6	56.4 ± 14.2
Sex (female %)	48 (48%)	26 (52%)	22 (44%)
BMI ≥ 30 (*n*, %)	29 (29%)	14 (28%)	15 (30%)
Duration of rheumatic heart disease (years, mean ± SD)	19.8 ± 16.2	20.4 ± 18.2	19.1 ± 14.1
EuroSCORE II (median [IQR])	3.8 [3.1–5.1]	4.7 [3.1–5.6]	3.8 [3.2–4.8]
LA diameter (cm, mean ± SD)	2.5 ± 0.4	2.5 ± 0.4	2.4 ± 0.3
Teichholz EF (%, mean ± SD)	52.2 ± 7.8	50.8 ± 8.7	53.6 ± 6.6
Simpson EF (%, mean ± SD)	49.0 ± 6.5	48.6 ± 7.3	49.5 ± 5.7
Hypertension (*n*, %)	96 (96%)	46 (92%)	50 (100%)
Diabetes mellitus (*n*, %)	26 (26%)	16 (32%)	10 (20%)
Stroke/TIA history (*n*, %)	3 (3%)	2 (4%)	1 (2%)
Ischemic heart disease, (*n*, %)	3 (3%)	3 (6%)	0 (0%)
Severity of disease (*n*, %)			
Moderate	81 (81%)	41 (82%)	40 (80%)
Severe	19 (19%)	9 (18%)	10 (20%)

ECG evaluation confirmed AF in all 100 patients, and the electrical axis of the heart (EAX) was recorded. Auscultation consistently revealed the presence of systolic murmurs in all participants. The majority of the patients (*n* = 81) were diagnosed with a moderate form of the disease, while others (*n* = 19) had a severe form.

### 3.2 Distribution of ablation and left atrial appendage occlusion methods

**Figure 2:**
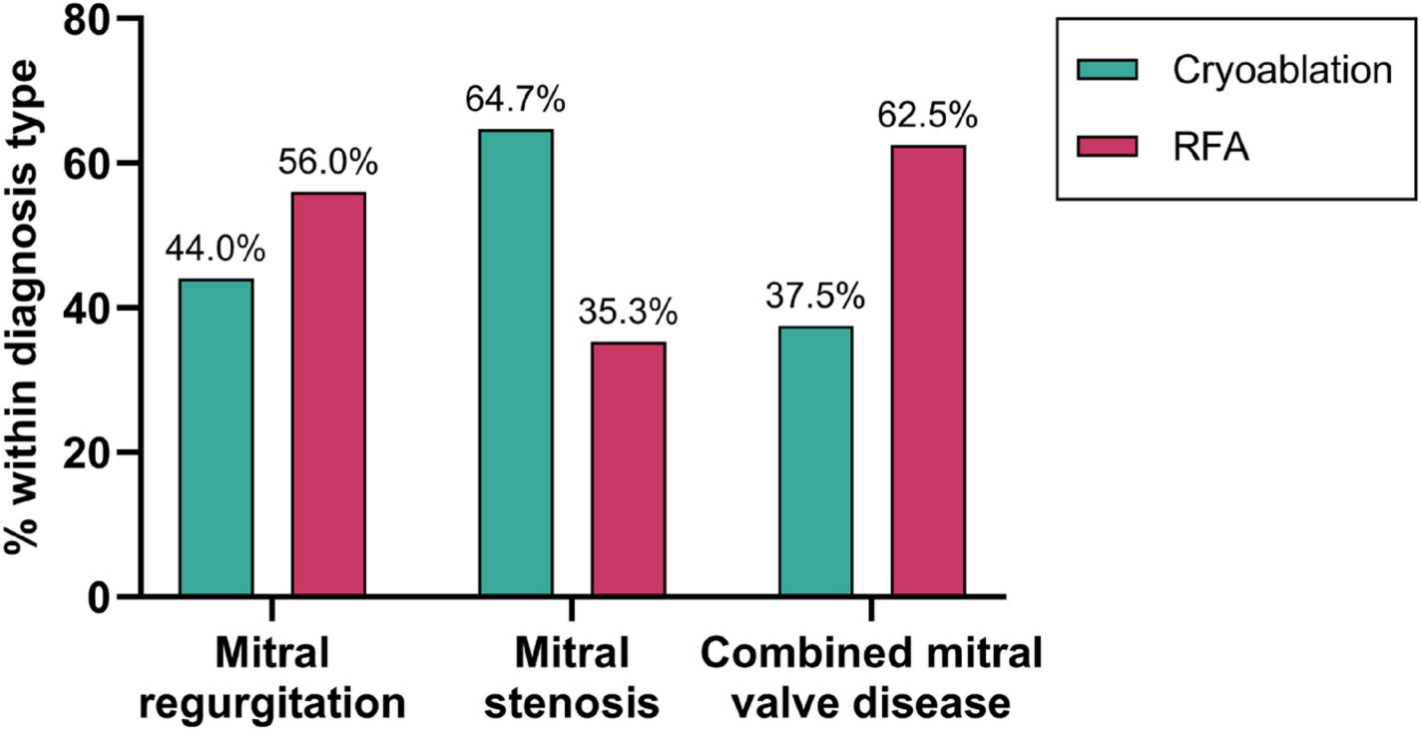
Distribution of ablation methods by diagnosis type. Percentages represent the proportion of patients within each diagnosis type (mitral regurgitation (*n* = 50), stenosis (*n* = 34), or combined valve disease (*n* = 16) who received either cryoablation or RFA.

The patients had three main diagnosis types: mitral regurgitation, mitral stenosis, or combined mitral valve disease. Cryoablation was more often performed in cases of mitral regurgitation and combined mitral valve disease. Whereas RFA was predominantly used in cases of mitral stenosis (Figure 2). There was no significant difference in the distribution of diagnosis types between the cryoablation and RFA groups (*p* = 0.097). Chronic rheumatic heart disease (CRD) was identified in all patients following surgical evaluation, underscoring its pivotal role in the pathogenesis of mitral valve disorders.

**Figure 3:**
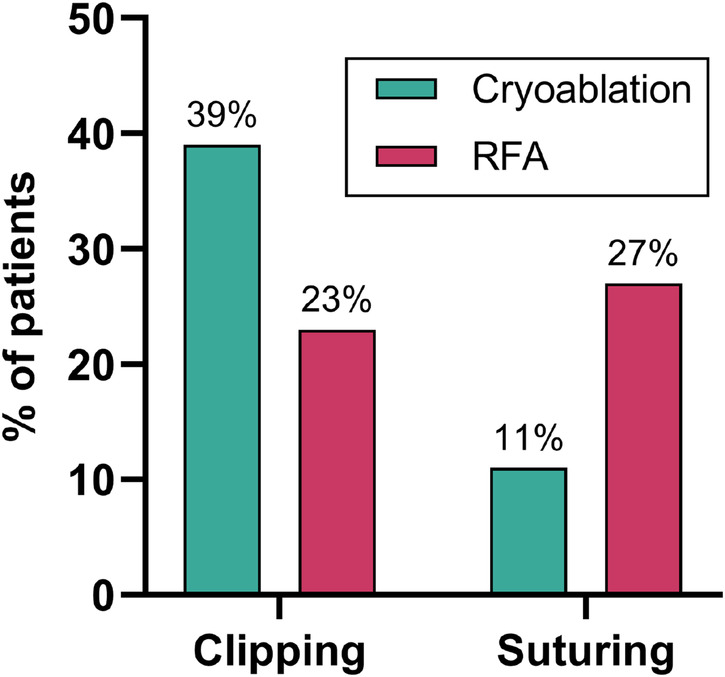
Comparison of LAA occlusion methods in cryoablation (*n* = 50) and RFA (*n* = 50) groups.

Surgical management of the left atrial appendage (LAA) was performed using two techniques: clipping (*n* = 61) and suturing (*n* = 39), depending on intraoperative findings and surgeon preference. There is a statistically significant difference between the cryoablation and RFA groups in the choice of the LAA occlusion method (*p* = 0.001). Clipping was more often used in the cryoablation group, and suturing was more often used in the RFA group (Figure 3).

### 3.3 Intraoperative parameters

All patients underwent mitral valve surgery with either annuloplasty repair or valve replacement (mechanical or bioprosthetic). Most prostheses were Medtronic, smaller numbers were from other manufacturers. No prosthetic dysfunction was observed postoperatively. Thrombectomy was performed in 8 cryoablation patients and 7 RFA patients (*p* = 0.681). Intraoperative parameters, CPB time, ACC time, blood loss, and total operation time, did not differ between groups (Table 2). However, the RFA group demonstrated a significantly higher mean heart rate compared to the cryoablation group (*p* = 0.007), indicating a potential difference in intraoperative autonomic or myocardial response.

**Table 2:** Comparison of intraoperative parameters between cryoablation and RFA groups.

Parameter	Cryoablation (*n* = 50)	RFA (*n* = 50)	*p*-Value
Heart rate (bpm, mean ± SD)	108.1 ± 32.1	120.2 ± 40.5	0.007
CPB time (min, mean ± SD)	139.6 ± 26.0	144.7 ± 24.3	0.318
ACC time (min, mean ± SD)	100.2 ± 24.0	96.7 ± 22.6	0.454
Blood loss (mL, mean ± SD)	212.2 ± 39.4	204.6 ± 37.0	0.285
Operation time (min, mean ± SD)	254.2 ± 40.2	245.5 ± 34.5	0.248

Intraoperative assessment revealed several structural abnormalities of the mitral valve that served as indications for surgical intervention. These included elevated transvalvular pressure gradients, leaflet thickening suggestive of fibrosis or calcification, restricted leaflet mobility indicating impaired valve function, and absence of counterphase motion, likely due to dystrophic changes or structural deformities. Mitral valve fibrosis was also frequently observed, reflecting progressive remodeling and reduced elasticity (Table 3). None of the structural mitral valve abnormalities differed significantly between groups.

**Table 3:** Mitral valve abnormalities in patients undergoing cryoablation and RFA.

Parameter	Cryoablation (*n* = 50)	RFA (*n* = 50)	*p*-Value
Mitral valve leaflets sealed (*n*)	47	50	0.242
Leaflet motion restriction (*n*)	26	22	0.423
Loss of counterphase motion (*n*)	16	8	0.061
Fibrosis of valve leaflets (*n*)	46	40	0.148

### 3.4 Pre- and postoperative clinical symptoms

All patients reported preoperative symptoms of fatigue, shortness of breath, and swelling of the lower extremities. Anginal pain was noted in 28% of patients in the cryoablation group and 22% in the RFA group. Following surgery, all clinical symptoms resolved completely in both groups, demonstrating the effectiveness of the surgical approaches in relieving symptoms of heart failure.

**Figure 4:**
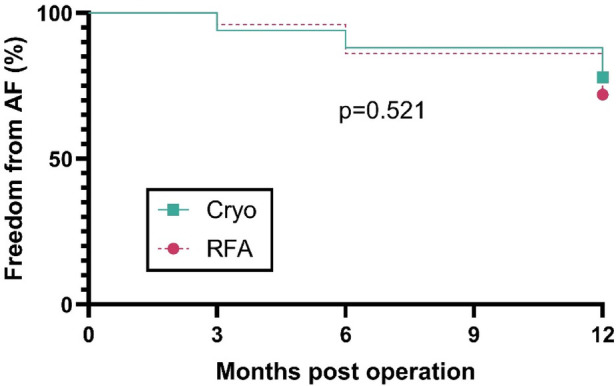
Freedom from AF after concomitant surgical ablation. Kaplan–Meier curves comparing cryoablation (*n* = 50) and RFA (*n* = 50) for time to first AF after mitral surgery. Follow-up assessments were performed at 3, 6, and 12 months post-operation. The *y*-axis shows the percentage of patients free from AF. Curves were compared with the log-rank test (*p* = 0.521).

Among 100 patients (cryo *n* = 50; RFA *n* = 50), first atrial arrhythmia recurrence occurred in 11 (22%) after cryoablation and 14 (28%) after RFA during follow-up. Kaplan–Meier curves for freedom from AF did not differ between groups (log-rank *p* = 0.521; Figure 4). The estimated hazard ratio for cryo vs. RFA was 0.78 (95% CI: 0.36–1.71; Mantel–Haenszel estimate: 0.76, 95% CI: 0.34–1.74), indicating no detectable difference in recurrence risk. Median time to recurrence was not reached in either cohort because >50% remained event-free.

### 3.5 Postoperative cardiac remodeling and electrophysiological outcomes

Immediate post-operative changes in most echocardiographic and electrophysiological parameters were similar between groups. Five measurements showed statistically significant differences. Left atrial (LA) volume decreased significantly more after cryoablation compared with RFA (*p* < 0.001). Multiple linear regression was performed to identify independent predictors of left atrial (LA) volume reduction at six months. The model was significant [*F*(5,94) = 3.95, *p* = 0.0027; *R*^2^ = 0.17]. Among all covariates, only ablation type remained an independent predictor (*β* = 27.88 ± 6.41 mL, 95% CI: 15.15–40.61; *p* < 0.0001), indicating that cryoablation was associated with a greater LA volume reduction compared with RFA. AF recurrence (*β* = 2.15, 95% CI: −11.4 to 15.7; *p* = 0.75), disease duration, BMI, and EuroSCORE II were not significantly associated with the degree of remodeling. Residuals deviated from normality, but multicollinearity was low (all VIF < 1.5) (Supplementary Table 1).

Aortic root diameter increased slightly following cryoablation but decreased after RFA (*p* = 0.023). The interventricular septum became thinner in the cryoablation group, but thicker in the RFA group (*p* = 0.026). Left ventricular (LV) posterior wall thickness decreased after cryoablation and increased after RFA (*p* = 0.018). Right atrial (RA) short-axis diameter showed a smaller reduction after cryoablation than after RFA (*p* = 0.040). All other parameters, including functional capacity (6-min walk distance), ventricular volumes, ejection fractions, pulmonary artery pressure, stroke volume, rhythm metrics (RR and QT intervals, heart-rate measures), and ΔC, did not differ significantly between the two groups (all *p* > 0.05) (Supplementary Table 2).

At six months after the operation, most echocardiographic and electrophysiological measures remained similar between the two groups, but three parameters still differed significantly. LA volume continued to decrease more after cryoablation than after RFA (*p* = 0.00004). RA short-axis diameter showed a smaller long-term reduction in the cryoablation group vs. the RFA group (*p* = 0.049). Stroke volume decreased slightly after cryoablation by only 1.22 ± 17.34 mL, but became significantly smaller by 7.28 ± 15.84 mL in the RFA group (*p* = 0.006). All other domains, including atrial and ventricular dimensions, aortic diameters, ventricular rates and intervals, contractile indices, pulmonary-artery pressure, and ΔC, showed no significant differences between groups at six months (all *p* > 0.05), (Supplementary Table 3).

## 4 Discussion

This retrospective cohort study compared cryoablation and radiofrequency ablation in patients with rheumatic mitral valve disease undergoing concomitant mitral valve surgery. Cryoablation was associated with a significantly greater reduction in left atrial volume, a smaller decrease in right atrial short-axis diameter, and a lesser decline in stroke volume compared with radiofrequency ablation. Procedural parameters, including cardiopulmonary bypass time, aortic cross-clamp time, and blood loss, were comparable between groups, and clinical symptoms improved similarly.

Although both strategies were derived from the extended modified Cox–Maze IV concept and targeted key arrhythmogenic substrates, the detailed lesion-set configuration differed between cryoablation and RFA in accordance with energy-specific surgical protocols. Therefore, the observed differences in atrial remodeling likely reflect the combined effect of lesion delivery characteristics and energy modality rather than fundamentally different ablation strategies.

The greater left atrial volume reduction observed with cryoablation may reflect biophysical differences between freezing and heating during lesion formation. Multiple linear regression analysis indicated that ablation modality, rather than perioperative factors or rhythm outcome, was the primary determinant of atrial reverse remodeling. Cryothermal energy produces homogeneous, transmural lesions with minimal collateral tissue edema, potentially facilitating more effective atrial shrinkage and remodeling (22, 23). In contrast, radiofrequency ablation relies on resistive and conductive heating, which may result in heterogeneous lesion thickness and surrounding tissue edema, potentially limiting structural reverse remodeling (24, 25).

Our findings are concordant with prior surgical series and meta-analyses reporting comparable rhythm outcomes between cryothermal and radiofrequency energy when ablation accompanies mitral valve surgery (11, 12, 14, 15, 19, 26, 27). In rheumatic mitral valve disease, where atrial fibrosis and remodeling are often advanced, previous studies have demonstrated that concomitant surgical ablation improves rhythm and clinical outcomes compared with valve surgery alone and that the choice of energy source does not independently compromise safety.

Despite differences in atrial remodeling, intraoperative safety and efficiency were equivalent across both groups. The absence of significant differences in cardiopulmonary bypass and aortic cross-clamp durations is consistent with contemporary surgical evidence indicating that energy selection does not materially alter operative workflow during concomitant AF ablation in mitral procedures (11, 24, 28).

An additional finding was the difference in left atrial appendage occlusion technique between groups: clipping predominated in the cryoablation group, whereas suturing was more frequent following radiofrequency ablation (*p* = 0.001). This pattern likely reflects surgeon preference influenced by intraoperative conditions and procedural workflow. Although epicardial clipping may offer improved stroke prophylaxis compared with suturing (29), this study was not designed to evaluate long-term thromboembolic outcomes, and this imbalance represents a potential confounder.

Several limitations warrant consideration. The retrospective, multicenter design and non-randomized sequential allocation introduce potential selection bias and confounding by indication. Lesion-set geometry differed between cryoablation and radiofrequency strategies, precluding attribution of observed effects solely to energy modality. The six-month follow-up captures early structural remodeling but does not assess late arrhythmia recurrence, survival, or stroke, and lesion transmurality or perilesional edema were not directly evaluated using advanced imaging. In addition, right-atrial lesion geometry differed substantially between groups, particularly with respect to cavocaval and coronary sinus–directed lines in the RFA cohort, which may have influenced right-atrial remodeling outcomes.

Future prospective randomized trials comparing cryoablation and radiofrequency ablation specifically in rheumatic mitral valve populations are required to validate these observations and assess long-term clinical outcomes. Incorporation of advanced imaging, such as late gadolinium enhancement MRI, to characterize lesion integrity and atrial fibrosis may further optimize patient selection and procedural strategy (30).

In conclusion, in patients with persistent atrial fibrillation and rheumatic mitral valve disease undergoing mitral valve surgery, cryoablation was associated with more favorable early atrial reverse remodeling than radiofrequency ablation without compromising procedural safety or efficiency. These findings should be interpreted as hypothesis-generating and require confirmation in randomized studies with standardized lesion sets and extended follow-up.

## Data Availability

The datasets used and/or analysed during the current study are available from the corresponding author on reasonable request. Requests to access these datasets should be directed to Zhanar Nurbay, nurbay2582@gmail.com.
