# Supplementary material for: Cryoablation and radiofrequency ablation during mitral valve surgery for rheumatic mitral valve disease: a retrospective cohort study
Source: Front Cardiovasc Med. 2026 Jan 22;12:1659310. doi: 10.3389/fcvm.2025.1659310 (PMC12872882; doi:10.3389/fcvm.2025.1659310)
Supplement: Supplementary file 1 [file Table1.docx]

***Supplementary Table 1.*** *Comparison of multiple linear regression models for predictors of LA volume reduction.*

| ***Variable*** | ***β (95% CI)*** | ***p-value*** |
| --- | --- | --- |
| Ablation Type (Cryo vs RFA) | +27.5 (15.1 to 39.9) | <0.0001 |
| Disease Duration (years) | +0.09 (–0.30 to 0.48) | 0.64 |
| BMI (kg/m²) | –0.56 (–1.75 to 0.62) | 0.34 |
| EuroSCORE II | +0.19 (–3.64 to 4.02) | 0.92 |
| AF Recurrence | +2.15 (–11.4 to 15.7) | 0.75 |
| Model statistics | F(4,95)=4.96, p=0.0011 | R²=0.173 |
| Multicollinearity (VIF) | All < 1.5 | — |
| Normality of residuals | Not normal (p<0.0001) | — |
