# Supplementary material for: Cryoablation and radiofrequency ablation during mitral valve surgery for rheumatic mitral valve disease: a retrospective cohort study
Source: Front Cardiovasc Med. 2026 Jan 22;12:1659310. doi: 10.3389/fcvm.2025.1659310 (PMC12872882; doi:10.3389/fcvm.2025.1659310)
Supplement: Supplementary file 3 [file Table3.docx]

***Supplementary Table 3.*** *Six-Month postoperative changes in cardiac structure and electrophysiological parameters following cryoablation versus RFA*

| **Domain** | **Parameter** | **Δ Cryo (mean ± SD)** | **Δ RFA (mean ± SD)** | **p-value** |
| --- | --- | --- | --- | --- |
| **Atrial remodeling** | LA volume (mL) | –50.16 ± 37.19 | –22.88 ± 13.67 | <0.001 |
|  | LA long-axis (cm) | –0.99 ± 0.70 | –0.77 ± 0.40 | 0.054 |
|  | LA short-axis (cm) | –0.50 ± 0.39 | –0.44 ± 0.38 | 0.197 |
|  | RA long-axis (cm) | –0.74 ± 0.60 | –0.64 ± 0.51 | 0.063 |
|  | RA short-axis (cm) | –0.47 ± 0.73 | –0.77 ± 0.71 | 0.049 |
| **Aortic geometry** | Aortic root diameter (cm) | 0.05 ± 0.32 | –0.07 ± 0.18 | 0.065 |
|  | Ascending aorta diameter (cm) | –0.02 ± 0.17 | 0.02 ± 0.20 | 0.674 |
| **Right ventricle** | RV diameter (cm) | –0.25 ± 0.94 | 0.06 ± 0.80 | 0.054 |
| **Rhythm metrics** | RR interval (ms) | –146.61 ± 514.63 | –25.38 ± 472.25 | 0.270 |
|  | QT interval (ms) | –0.71 ± 103.86 | –4.20 ± 50.52 | 0.161 |
|  | Avg. heart rate (bpm) | –18.34 ± 28.75 | –29.28 ± 27.72 | 0.098 |
| **Contractile function** | Teichholz EF (%) | 0.06 ± 11.14 | –1.86 ± 9.00 | 0.170 |
|  | Simpson EF (%) | 0.34 ± 7.74 | 0.42 ± 7.73 | 0.480 |
|  | Stroke volume (mL) | –1.22 ± 17.34 | –7.28 ± 15.84 | 0.006 |
| **Ventricular structure** | RV diameter (cm) | –0.25 ± 0.94 | 0.06 ± 0.80 | 0.054 |
|  | LV diastolic diameter (cm) | –0.19 ± 0.69 | –0.14 ± 0.69 | 0.802 |
|  | LV systolic diameter (cm) | –0.16 ± 0.68 | –0.13 ± 0.78 | 0.495 |
|  | LV diastolic volume (mL) | –11.36 ± 42.40 | –15.52 ± 32.34 | 0.530 |
|  | LV systolic volume (mL) | –11.76 ± 47.34 | –4.40 ± 17.94 | 0.444 |
|  | Interventricular septum (cm) | –0.05 ± 0.16 | 0.02 ± 0.15 | 0.512 |
|  | LV posterior wall (cm) | –0.02 ± 0.14 | 0.05 ± 0.15 | 0.067 |
| **Hemodynamics** | Δ C (%) | –0.02 ± 5.54 | –0.48 ± 3.55 | 0.822 |
|  | Estimated PASP (mm Hg) | –13.87 ± 7.10 | –13.02 ± 5.88 | 0.952 |
